# Data Collection and Analysis Using Wearable Sensors for Monitoring Knee Range of Motion after Total Knee Arthroplasty

**DOI:** 10.3390/s17020418

**Published:** 2017-02-22

**Authors:** Chih-Yen Chiang, Kun-Hui Chen, Kai-Chun Liu, Steen Jun-Ping Hsu, Chia-Tai Chan

**Affiliations:** 1 Department of Biomedical Engineering, National Yang-Ming University, No. 155, Li-Nong St., Section 2, Peitou, Taipei 11221, Taiwan; phinyen@gmail.com (C.-Y.C.); khc@vghtc.gov.tw (K.-H.C.); g30104026@ym.edu.tw (K.-C.L.); 2 Department of Orthopaedic Surgery, Taichung Veterans General Hospital, 1650 Taiwan Boulevard Section 4, Taichung 40705, Taiwan; 3 Department of Information Management, Minghsin University of Science and Technology, No.1, Xinxing Road, Hsinchu 30401, Taiwan; steenhsu@must.edu.tw

**Keywords:** inertial sensor, wearable sensor, total knee arthroplasty, TKA, goniometer, range of motion, ROM

## Abstract

Total knee arthroplasty (TKA) is the most common treatment for degenerative osteoarthritis of that articulation. However, either in rehabilitation clinics or in hospital wards, the knee range of motion (ROM) can currently only be assessed using a goniometer. In order to provide continuous and objective measurements of knee ROM, we propose the use of wearable inertial sensors to record the knee ROM during the recovery progress. Digitalized and objective data can assist the surgeons to control the recovery status and flexibly adjust rehabilitation programs during the early acute inpatient stage. The more knee flexion ROM regained during the early inpatient period, the better the long-term knee recovery will be and the sooner early discharge can be achieved. The results of this work show that the proposed wearable sensor approach can provide an alternative for continuous monitoring and objective assessment of knee ROM recovery progress for TKA patients compared to the traditional goniometer measurements.

## 1. Introduction

Osteoarthritis of the knee is a degenerative disease that greatly impacts the activities of daily living (ADLs) and the quality of life of the elderly. The most common treatment method is Total Knee Arthroplasty (TKA). By using artificial implants to replace the damaged knee joint, the knee functions can be regained. In the United States, nearly 1.5% of Americans are living with a knee replacement, and for people aged over 50 years old the prevalence of TKA is up to 4.6% [1]. More than 700,000 TKA procedures are performed annually in the US, and the number of TKA procedures is predicted to increase greatly up to 3.48 million by 2030 due to the expected TKA revisions [2], although in general, patients requiring revision surgery in 10 years is less than 5% and it is very low overall [3].

The most important goal of TKA is to obtain postoperatively a maximized and function-regained range of motion (ROM) of the knee joint. However, during their recovery many TKA patients suffer from knee stiffness and reduced knee flexion of less than 90°. Indeed, restricted postoperative knee flexion is the most frequent complication after TKA procedures and it is also the main cause of patient dissatisfaction [4]. The recommended knee ROM recovery for performing ADLs is at least 100° of knee flexion. Tasks such as walking up stairs require at least 83° of knee flexion, 90° to 100° are needed for walking down the stairs, 93° to 105° to arise from a chair, and more than 115° to squat or kneel [5,6]. 

Naylor et al. found that discharge knee ROM may be a useful clinical indicator to evaluate long-term knee ROM restoration prognosis and it may be important for physiotherapists to maximize knee ROM in the early and sub-acute stages after TKA procedures [7]. Restoration of postoperative knee ROM affects both the patient’s health and medical service expenditures. Patients who have difficulties in regaining the knee ROM must be discovered early and provided with more health services and direct assistance [8]. Of interest to these studies [5,6,7,8], active knee flexion recovery before hospital discharge has revealed a correlation with knee ROM at 12 months postoperation [7,8]. This would suggest that if patients can improve their knee flexion during the early acute inpatient stage, long-term health outcomes and early discharge will be beneficial to the patients.

Aggressive knee rehabilitation programs enable patients to restore their functional capability to normal, so that they can obtain better recovery of long-term knee ROM and early discharge as soon as possible. To achieve this target, continuous monitoring of postoperative knee ROM, in-time correction during rehabilitation programs, and flexible adjustment of the rehabilitation force are required. The purpose on continuous monitoring is to collect as much data as possible so as to control the recovery conditions for postoperative TKA and do the necessary adjustments for the patients. In this study, we aimed to propose a method to conduct the data collection and data analysis for monitoring the recovery progress of knee ROM on TKA patients by using wearable sensors. 

The rest of this paper is organized as follows: Section 2 briefly introduces related works and approaches. Section 3 then details the proposed system architecture and the data handling methods for the collected data. Section 4 demonstrates the real data collected from patients and the analysis results. Finally, Section 5 discusses the results, concludes the work, and addresses the future tasks of this study. 

## 2. Related Works

The human skeleton is a highly articulated structure that moves based on the twists and rotations between the bone and the joints. It requires a very high degree of freedom for motor activity [8]. Many methods for analyzing the range of motion of the human skeleton have been proposed. This section reviews the literature on technologies and methods involved in motion tracking for rehabilitation and gait analysis. In the field of traditional biomechanical engineering, gait cycle analysis is the most interesting topic for researchers. Studies focused on the motions of specific segments of the human skeleton, such as knees or shoulders, are relatively rare. This section introduces the related works in two subsections. 

### 2.1. Progress of Measurement-From Subjective to Objective

With the advancement of technology, it is obvious that motion tracking of human skeletons has progressed from subjective to objective. Among traditional ways of monitoring range of motion, visual-based tracking and goniometers are the most common methods. However, in these monitoring methods it is difficult to avoid the individual offsets resulting from human judgement.

Lavernia et al. conducted a survey to evaluate the fairness and objectiveness by comparing ROM measurements using goniometers [9]. This study took radiographic measurements as the gold standard and compares the results of goniometer measurements performed by professionals with five different roles. The results showed that higher ROM might be overestimated and measurement differences exist between different individuals. Though radiographs are the gold standard for measuring knee joint flexion/extension angles, goniometers are more convenient to use in the clinic and they cause no radiation exposure. On the negative side, measurement offsets caused by the human eye’s subjective judgement are inevitable.

Objective measurements and meaningful quantitative methods are two critical requirements for motion tracking. The most valuable approach is one able to distinguish tiny variations in knee ROM and free from the interference of pain. Boonstra et al. proposed two quantitative approaches in 2008, one is patient-based questionnaires and another is performance-based tests [5,10]. This team proposed an indicator called WOMAC osteoarthritis index to evaluate the patient's physical performance in the postoperative stage. Although this approach is objective and quantitative, it can only obtain the outcomes of postoperative patients without the improvement of recovery records and the data of knee/hip ROM.

Huosheng et al. classified human motion tracking systems into three types, which are visual tracking, non-visual tracking, and robot-aided tracking [8]. The visual-based motion tracking systems may include cameras, reflective tracking markers, feature extraction techniques and imaging processing techniques. However, the visual tracking systems might be locally restricted and expensive. It is also difficult to monitor different patients continuously. Non-visual motion tracking systems mostly use sensors for monitoring [8], such as inertial sensors, magnetic sensors, and sensor-integrated gloves [11,12,13,14,15,16,17,18]. The digital data collected by the sensors is considered to be a useful tool for monitoring postoperative patients continuously. In the past five years, many sensor-based studies were proposed for motion tracking and gait analysis. The related works will be discussed in the next section. Robot-aided tracking systems use robots to support and control limb motions by incorporating many sensors to achieve sense-measure-feedback mechanism. Although such a system may be helpful to postoperative patients, either on supporting the postoperative limbs or monitoring the recovery of the limbs, the incorporation of the rehabilitation robots and sensors makes this a big and often very expensive system, which is not suitable for minimally invasive surgeries, such as TKA because most TKA patients will have early in-patient rehabilitation programs and the sensor measurements will be interfered with by the robot supporting system.

From the above related works, the utility of using sensors for monitoring human motions is quite apparent. Cost effective and easy deployment allow for multiple sensors to be mounted collectively on different body segments. Synchronized monitoring of multiple body segments had drawn many studies falling into a similar infrastructure. We call this skeleton-based monitoring infrastructure. In Section 2.2, related studies on knee ROM and gait analysis are introduced. 

### 2.2. Skeleton-Based Monitoring Infrastructure

Many studies have monitored motions and analyzed each body segment to assess the ROM and ambulatory performance. As listed in Table 1, these works have developed their own analysis approaches by using different sensor combinations, but they were all based on the motion analysis of each skeleton segment. 

Basically, the principle is to wear at least two sensor units on two body segments and to calculate the ROM by taking the joint as a fulcrum. As for the gait analysis, it undoubtedly requires more sensor units. In general, such approaches are widely accepted and persuasive. Currently surgeons can only use goniometers to obtain the postoperative knee ROM during routine ward inspections or during clinic visits for outpatients. In recent years, many researchers had proposed many novel systems using sensors [8,9,10,11,12,13,14,15,16] for monitoring recovery conditions for postoperative TKA patients. 

Although motion sensors are highly sensitive and can be used in a wide range of fields, they have some disadvantages. For example, the recordings might have fluctuations induced by noises and poor adhesion problems [19]. All these issues will lead to sensor drift problems. Several calibration protocols were proposed for knee joint rehabilitation, and the method proposed by Baker in 2006 [20] is so far still adopted as a gold standard [21]. In 2009, Takeda et al. used wearable gyro and acceleration sensors to estimate postures [22]. They used a mechanical turntable to initialize the zero offset of accelerations and angular data from the sensors. Caroselli et al. proposed a very simple architecture to estimate the angular kinematics of body segment movement [23]. Their results showed that single-axis accelerometers can measure comparable angular data to the mechanical pendulum. From these studies, two alternatives can be suggested for solving the sensor drift problem. One is to apply external corrections before using these sensors, such as using the mechanical pendulum [23]. Another method is to use other available sensors for compensation, cross validation and correction, such as by designing a mechanical turntable or a synchronized reset button [22].

From the above literatures, studies that focused on measuring the knee ROM is relatively rare compared to gait cycle studies. Although the knee ROM seems to be part of gait analysis, there are only a few works proposing effective approaches for knee ROM calculation. In this work, we adopted the skeleton-based infrastructure. The knee ROM estimation refers to the methods proposed by Takeda et al. [13]. However, the methods for long-term knee recovery monitoring require further discussion. In Section 2.3, we will present our research roadmap and explain the requirements for such long-term monitoring.

### 2.3. Motivations of This Study

According to the standard of care for TKA proposed by Brigham and Women’s Hospital (BWH) [24], the major objective is to aid the patients return to normal biomechanical status and improve their knee ROM. Following the guidelines in the standard, caring acts can help the patients to recover their muscle balance ability, to alleviate their pain, and to improve the knee function and the quality of life. In this study, we are working on the same goals as this standard. 

In our previous work [18], we proposed a rehabilitation monitoring mechanism for long-term outpatient/home TKA recovery. This study used feature extraction to process the collected data and applied different classifiers to identify the rehabilitation activities. In another study [25], we proposed a system architecture for TKA patients at the acute inpatient stage. We conducted small scale experiments on four healthy subjects and five TKA patients to verify the proposed method. The results showed that the monitoring mechanism reveals that the knee ROM can be restored to the initial level before surgery. 

In this study, we followed the same experimental design that we proposed in [25]. We attempted to conduct a large scale clinical investigation on TKA patients for the purpose of improving wearable sensor use and further analysis including the patients’ personal factors, such as anesthesia status, hemostatic agents, and BMI value. In this work, we conducted the monitoring on 18 TKA patients and also prepared a questionnaire to survey the improvements for comfortable sensor-worn methods. The experiments will be introduced in Section 3.

## 3. Materials and Methods

**Figure 1:**
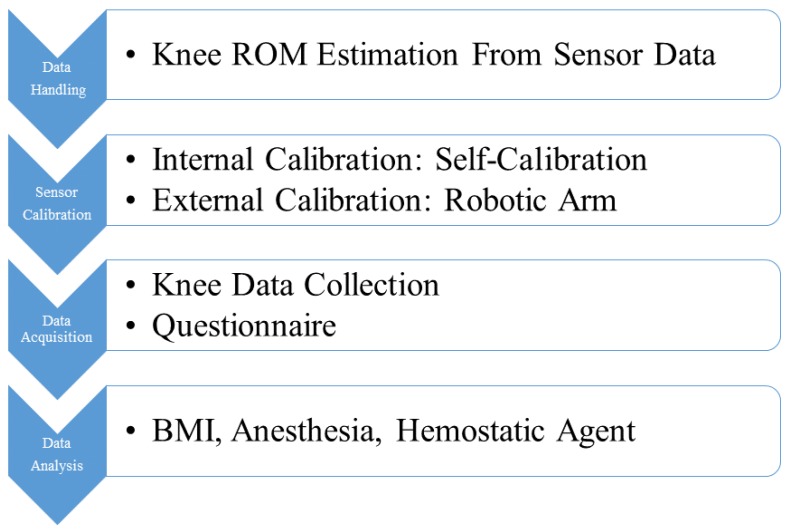
The workflow and tasks of this study.

The workflow and tasks of this study are depicted in Figure 1. According to the workflow, we divided this section into four subsections. The first subsection will describe how we estimate the knee ROM from the sensor recording. Section 3.2 will introduce the sensor calibration procedures before data collection. The third subsection will demonstrate the data collection experiments and the questionnaires in collecting the patient opinions on sensor-worn methods. The final subsection will introduce the data analysis targets including patient’s BMI, post-TKA anesthesia status, and the conditions using hemostatic agents.

### 3.1. Data Handling

This section demonstrates the data handling methods for the collected sensor measurements. The derivation for the estimation of knee ROM is going to be introduced in the following paragraphs. The correlation analysis between the knee ROM and three critical factors regarding the patient’s status will be introduced in Section 3.4. 

**Figure 2:**
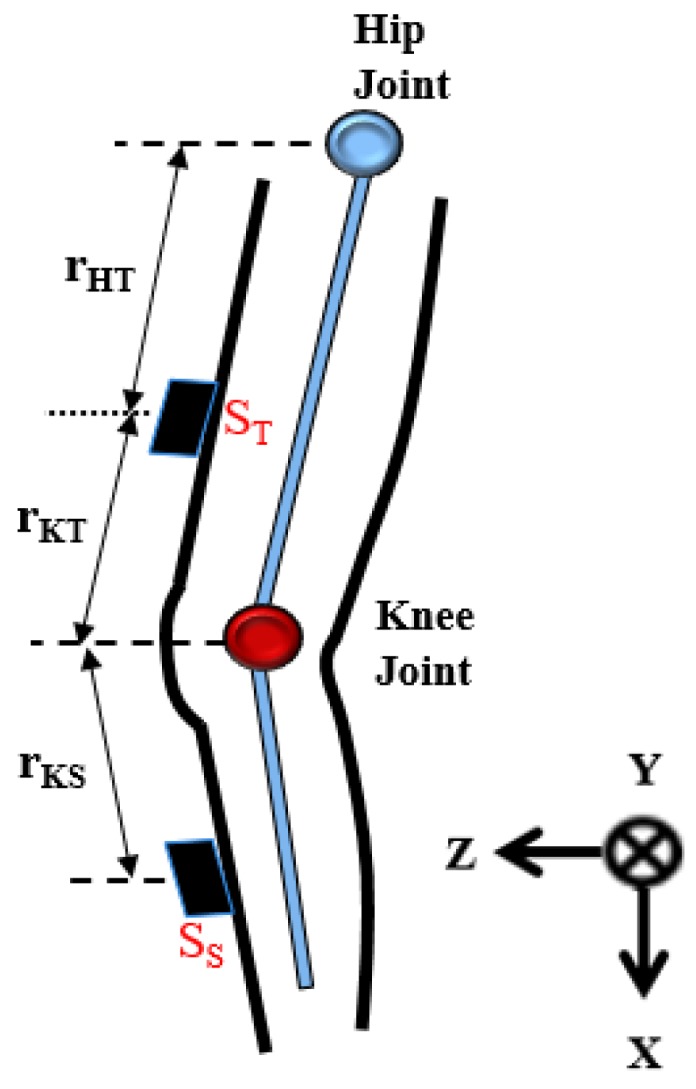
The coordinate system and the sensor model on the right leg. The X axis is the direction toward gravity, the Y axis is left-lateral direction and the Z axis is the walking direction. (S_T_ denotes a sensor on the thigh. S_S_ denotes a sensor on the shank. $r_{HT}$ means the distance from the hip joint to the thigh sensor. $r_{KT}$ means the distance from the knee joint to the thigh sensor, and $r_{KS}$ means the distance from the knee joint to the shank sensor).

As illustrated in Figure 2, the sensor distance to the knee joint and the inclination of each body segment were used to calculate the motion angles of the knee joint. The sensor nodes are placed on the thigh and the shank respectively (denoted as S_T_ and S_S_). Every sensor node placed on the body segment can record the accelerations of inclination of the thigh or the shank. The output of the sensor nodes can be divided into translational and gravitational components. The signals from accelerometers are used to calculate the angles of hip flexion and knee flexion.

Firstly, as it can measure the gravitational acceleration, the output of the sensor node $O_{i}$ can be expressed as:

(1) $$O_{i}=a_{i}+g_{i}$$

where $a_{i}$ is the translational acceleration and $g_{i}$ is the gravitational acceleration. The $a_{i}$ is 0 meaning that the sensor node is static and the gravitational acceleration is the only output of sensor node. Therefore, the joint angles α, β, and γ are the tilt angles along the x, y and z axis in the static state, which can be expressed as:

(2) $$\alpha =\tan^{-1}\left(\frac{A_{x}}{\sqrt{A_{y^{2}}+A_{z^{2}}}}\right)$$

(3) $$\beta =\tan^{-1}\left(\frac{A_{y}}{\sqrt{A_{x^{2}}+A_{z^{2}}}}\right)$$

(4) $$\gamma =\tan^{-1}\left(\frac{A_{z}}{\sqrt{A_{x^{2}}+A_{y^{2}}}}\right)$$

where $A_{x}$, $A_{y}$, and $A_{z}$ represent the tri-axial components of the gravitational acceleration respectively.

Secondly, the approach to gather the knee joint angle is based on Takeda et al. [14] work, which eliminates the acceleration components and keeps the gravitational components to calculate the inclination variation angles during movement. Two sensor nodes placed on the thigh segment and the shank are used to estimate the knee joint angle during knee flexion/extension. The translational acceleration $a_{T}$ can be expressed using the following equation:

(5) $$a_{T}=a_{K}+{\ddot{r}}_{KT}$$

where $a_{K}$ is the acceleration at knee joint, ${\ddot{r}}_{KT}$ is the rotational velocity from thigh to knee. Based on the accelerometer and gyroscope signals, the rotational velocity of the thigh ${\ddot{r}}_{KT}$ has two components, the centripetal ($\omega_{T}\times \left(\omega_{T}\times r_{KT}\right)$) and tangential acceleration (${\dot{\omega }}_{T}\times r_{KT}$), which can be expressed as:

(6) $${\ddot{r}}_{KT}={\dot{\omega }}_{T}\times r_{KT}+\omega_{T}\times \left(\omega_{T}\times r_{KT}\right)$$

where the ${\ddot{r}}_{KT}$ is rotational velocity of the thigh segment. ${\dot{\omega }}_{T}$ is the angular acceleration of the thigh segment. $r_{KT}$ is the distance from knee joint to the thigh sensor, and $\omega_{T}$ is the angular velocity of the thigh.

**Figure 3:**
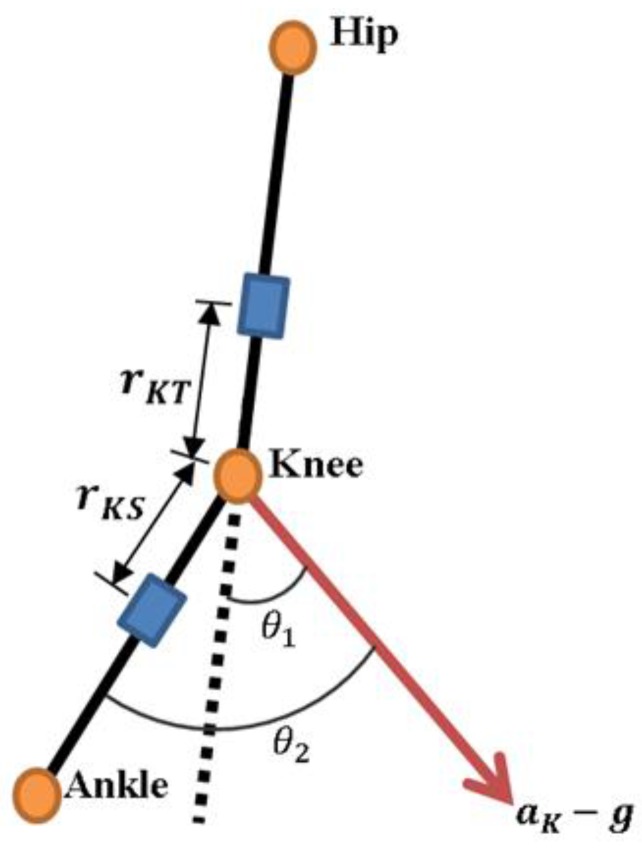
$\theta_{1}$ is the inclined angle of $a_{K}-g$ in relation to thigh segment and $\theta_{2}$ the inclined angle of $a_{K}-g$ in relation to shank segment.

As shown in Figure 3, $\theta_{1}$ is the inclination angle of $a_{K}-g$ in relation to the thigh segment and $\theta_{2}$ the inclination angle of $a_{K}-g$ in relation to the shank segment. The values of $\theta_{1}$ and $\theta_{2}$ can be calculated by the following equations:

(7) $$\;\theta_{1}=\tan^{-1}\frac{{\left|O_{T}-{\ddot{r}}_{KT}\right|}_{z}}{{\left|O_{T}-{\ddot{r}}_{KT}\right|}_{x}}$$

(8) $$\;\theta_{2}=\tan^{-1}\frac{{\left|O_{S}-{\ddot{r}}_{KS}\right|}_{z}}{{\left|O_{S}-{\ddot{r}}_{KS}\right|}_{x}}$$

Finally, the knee joint angle during the knee flexion-extension φ can be obtained by the difference between $\theta_{2}$ and $\theta_{1}$, as shown in the following equation:

(9) $$\varphi_{K}=\;\theta_{2}-\;\theta_{1}$$

### 3.2. Sensor Calibration

Before real data collection in a clinical environment, the zero drift issues of the sensors and the accuracy verification must be dealt with in advance. Therefore, the most important step is to calibrate the sensors prior to their use on the patients. Inaccurate recordings will result in analysis bias and the loss of clinical samples for which the clinical data is often rare and valuable. The sensors can automatically perform self-calibration through the software user interface (UI) provided by the sensor systems. Through the software UI, each sensor, gyroscope or accelerometer, can be calibrated separately or just returned to its factory settings. 

**Figure 4:**
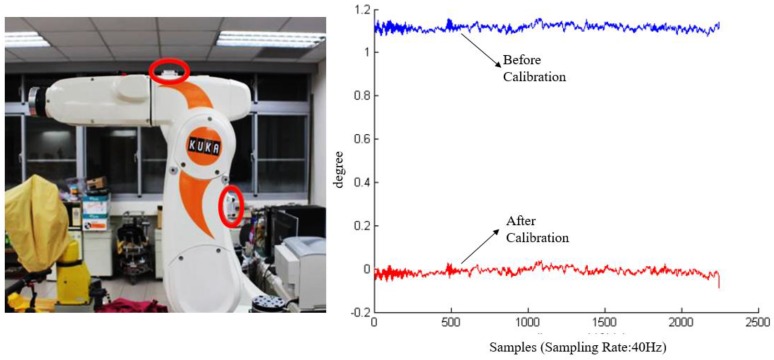
The robotic arm for sensor calibration.

After the internal calibration was completed, we used a robotic arm as a standard tool to cross check the sensor accuracy. The robotic arm (KR 5 sixx R650, KUKA, Singapur, Singapore) has 6-axis degree of freedom in movements and a repeatability accuracy of up to ±0.02 mm [26]. The advantage in using a robotic arm is the sensors can be placed on the two arms so as to simulate the real conditions of sensors worn on the thigh and the shank. As shown in Figure 4, the two red circles indicate the locations of two sensor nodes. The robotic arm can be configured to move and rotate along a specified path or angle, therefore the sensor recordings can be compared with the designated movement path of the robotic arm.

As shown in the left picture in Figure 4, the upper arm of the robot is parallel to the horizontal plane in the initial state. The movement angle at the initial position is defined as zero. We configured the arm to move 90° clockwise and then move back to the initial position. This is defined as one movement cycle. During an external calibration, the arm will perform the movement cycle ten times so as to collect sufficient sensor data for calibration. Then the offsets can be eliminated as shown in the right picture in Figure 4.

### 3.3. Data Acquisition

After calibration, the sensors are ready to monitor the TKA patient’s knee ROM. In this work, we recruited 18 TKA patients and collected their knee data four times before and after TKA. The four collections were before the TKA procedure, two days after TKA, two weeks after TKA, and six weeks after TKA. At the final data collection, a questionnaire was issued to collect the patients’ opinions regarding their sensor wearing experience, with the purpose of identfying possible improvements for sensor-worn methods. 

#### 3.3.1. Knee Data Collection

**Figure 5:**
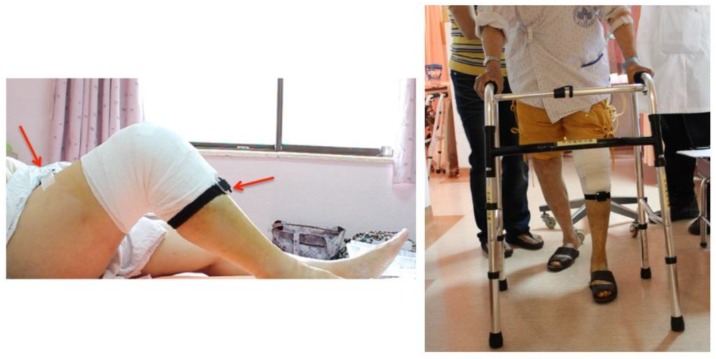
Two sensors were mounted on the anterior side of the thigh and shank.

The sensory system we used for data acquisition is called the APDM movement monitoring system. Each APDM sensor node includes an accelerometer, gyroscope, barometer, magnetometer, and temperature sensor. In this work, we enabled only the recordings of accelerometer and gyroscope for two reasons. One of the main purposes was to save power since continuous measurements are quite power-consuming. Another reason was to reduce the interferences from the environment to a minimum. For example, the magnetometer can easily suffer interferences from many magnetic forces and ferrite-based equipment. Two APDM sensors were mounted on the anterior surface of the patient’s thigh and shank of the surgical limb, as shown in Figure 5. A sensor node is 48.5 × 36.5 × 13.5 mm^3^, weighs 22 g. The sampling rate is 40 Hz. The measurement range of the accelerometer is ±58.5 m/s^2^ (6 g). The X and Y axis of the gyroscope have a range of ±34.9 rad/s, and the range of the Z-axis is ±26.8 rad/s. 

We established a monitoring protocol to collect the data in the hospital ward and in the outpatient clinic. Before using the wearable sensors, the medical professionals were trained and tested. The monitoring protocols for the medical professional were as follows:

(1) Take two calibrated sensors and record their serial numbers on the data collection table. Unless there are special requirements, the sensors can only monitor the surgical limb.
(2) After confirming that the green light of the sensor is on the upper left side, place two sensors on the patient's anterior thigh and shank of the surgical limb. It is recommended to mount the sensor 10 cm from the knee joint. The front side of the sensor should be kept parallel to the coronal plane of the human body.
(3) Measure the distance $r_{KT}$ (from the knee joint to the thigh sensor) and the distance $r_{KS}$ (from the knee joint to the shank sensor). Write down these values on the data collection table.
(4) Let the patient lie down and ask the patient to stretch the surgical limb on the bed. Then, while keeping the foot on the bed slide the foot from the distal to proximal body position so as to let the knee bend as much as possible (as shown in the left picture of Figure 5). Ask the patient to perform this task three times.
(5) Ask the patient to get off the bed and walk to the aisle. This task is to record the patient’s walking status from the first marking to the second marking on the floor. The distance is about ten meters. The patient should try to walk as normal as possible.
(6) If any of above tasks were not well performed, ask the patient to repeat that task again, remembering to erase the previous data and write down the notes clearly on the data collection table.

#### 3.3.2. Questionnaire

The sensor wearing methods have long been a common issue for most wearable sensor systems. The issues include patient discomfort, sensor displacement induced noises, and so on. For the purpose of improving the sensor-worn methods, we used a questionnaire to collect the patients’ opinions. The contents of the questionnaire were as follows:

(1) Do you feel any discomfort while wearing the sensors?
(2) Does the existence of the sensors influence your ambulatory and knee motions?
(3) Please tell the professionals about the discomfort induced by the sensors and how the sensors influence your normal gait and knee motion.
  Example: something on my lower limb that makes me uncomfortable.
  Example: the sensor belt is too tight that makes me unable to walk normally.
(4) Would you like to wear the sensors after discharge? Please note that the sensors and your surgical limb cannot touch any water during the monitoring period. The sensors will be taken off after two weeks in the outpatient clinic.
(5) If the sensors were worn using a water-proof securement dressing, would you prefer to wear the sensors? Please note that the sensors and your surgical limb cannot touch any water during the monitoring period. The sensors will be taken off after two weeks in the outpatient clinic.
(6) Please write down any additional comments or suggestions.

### 3.4. Data Analysis

In this work, we conducted a correlation analysis between the collected knee ROM and the factors regarding the patient's status. We selected three critical factors for this data analysis. The first one is the body mass index (BMI). This is the most direct and common factor since the patient’s weight is directly applied on the post-TKA knee. Patients’ ambulatory condition is almost always positively related to their BMI values [27]. The second factor is the patient’s anesthesia status. One of the most common pain relief methods is to use Epidural Patient Control Anesthesia (EPCA). Once the pain is relieved, patients might be willing to get off the bed and walk. The third factor is the hemostatic agents applied on the patients perioperatively. The major agents used are transamin or tranexmic acid. The purpose of using a hemostatic agent is to reduce the blood loss during the surgery. The use of hemostatic agents can be classified into two types. One is to provide the agents intravenously, and another one is to deliver the agents intra-articularly. The hemostatic agents might further affect the recovery conditions of the wounds [28]. This is why we choose this factor for analysis. 

## 4. Results

This section is divided into two subsections. Section 4.1 will present the data collection of 18 TKA patients. Four data collection sessions were conducted on each patient. The first data collection is performed before TKA surgery, while the remaining data collections ocurred at two days, two weeks, and six weeks after the TKA surgery. Section 4.2 summarizes the conclusions from the questionnaire with respect to the comments about the sensor-worn methods.

### 4.1. Knee ROM of 18 TKA Patients

**Figure 6:**
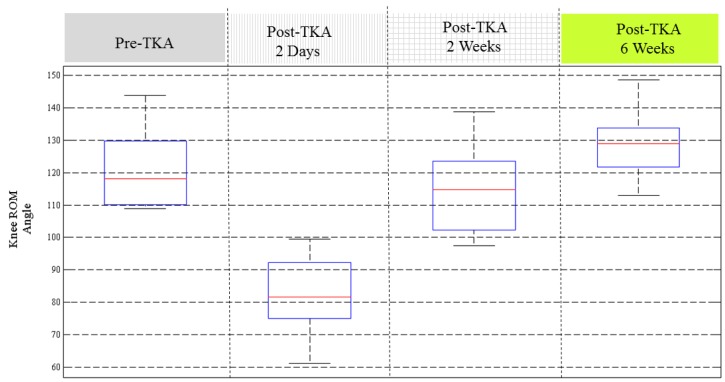
The recovery knee ROM angles collected from 18 TKA patients at four stages.

In this study, we conducted data collection and analysis on 18 TKA patients. Figure 6 illustrates the knee ROM recovery status of the 18 patients. The results show that most of the patients had recovered in two weeks after TKA. By six weeks after TKA, the knee ROM almost returns to the conditions before surgery. Some patients had better knee ROM performance two days after TKA, but some patients don’t have such symptoms. Some patients recovered with larger knee ROM at 6 weeks post-operation than before the TKA procedure. The results in Figure 6 indicate that there are certain critical impact factors related to the recovery of knee ROM. 

**Figure 7:**
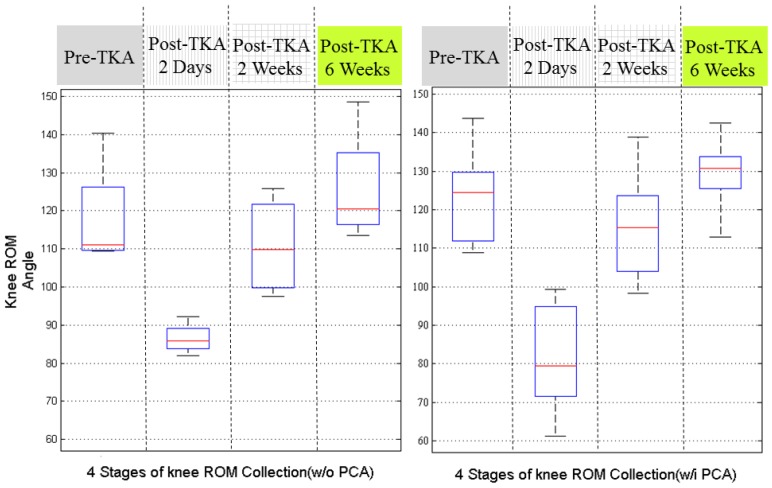
The recovery knee ROM between patients with and without Epidural PCA (EPCA).

Figure 7 illustrates the recovery status by dividing the 18 patients according to the use of EPCA. Before the analysis, we assumed that the patients with EPCA might have better knee ROM performance since their pains were alleviated. 

Unexpectedly, the results were not as we predicted, although during the longer recovery period, the patients who used EPCA seemed to have better knee ROM recovery at 2 weeks after surgery. These results are however not solid because the patient group without using EPCA originally had smaller knee ROM before TKA. Therefore, these results require further discussion.

**Figure 8:**
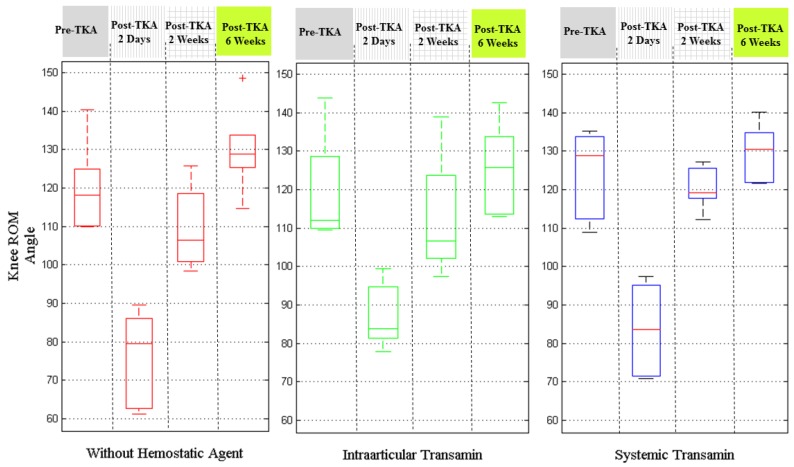
The recovery knee ROM between groups of patients differing in use of hemostatic agents.

Figure 8 depicts the results by the injection conditions of hemostatic agents. The patients with intra-articular transamin treatment seemed to have better knee ROM two days after surgery, particularly compared with those patients without using any hemostatic agent. The patients administered systemic transamin had the best knee ROM performance at 2 weeks and 6 weeks after their TKA procedures.

**Figure 9:**
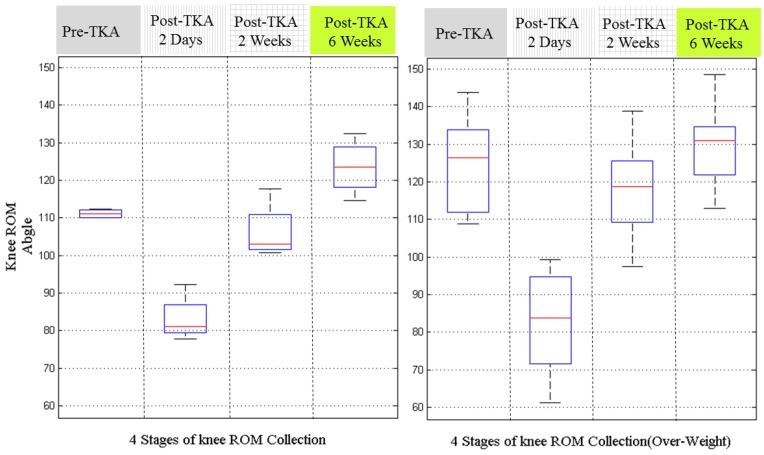
The recovery knee ROM between fat and thin patients (patients are grouped by BMI over and under 28).

Figure 9 is the results by grouping the patients with BMI values above or below 28. The patients with BMI over 28 is taken as the overweight group [27]. From the results, we can estimate that the thin group has better knee ROM recovery. However, these results are not solid either, because at pre-TKA stage, the knee ROMs of the thin group was better those of the fat group. This indicates the patients should be pre-grouped according to their pre-operative knee ROM.

### 4.2. Results of Questionnaires

**Figure 10:**
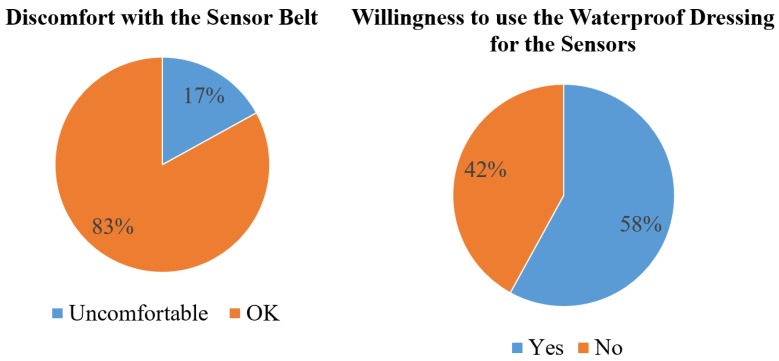
The results of questionnaire for surveying the sensor-worn scenario.

Figure 10 shows the results of the questionnaire. The first result is about the feelings for sensors worn by using a belt. Most of the patients felt OK, and only 17% patients felt uncomfortable. The second result is to understand the patient’s willingness if the belt were to be changed by using a waterproof dressing to stabilize the sensors. The results show that 58% patients would like to change the sensor wearing scenario by using the waterproof dressing. 

## 5. Discussion and Conclusions

In this work, we have proposed a monitoring protocol and an approach for the purpose of aiding the TKA patients and surgeons to control the recovery of knee ROM. The major contribution of this study is to provide an effective monitoring approach, and the future goal will be able to positively aid the TKA patients by adjusting the rehabilitation force based the monitoring feedback. The long-term goal is to introduce the monitoring mechanism for outpatients at home. 

In the proposed system architecture, the sensory system requires self-calibration and external calibration to confirm the accuracy of the sensors before data collection. The external calibration requires a robotic arm. This means the hospital might require dedicated personnel or a department to perform the external calibration and maintain the robotic arm. This is not realistic or convenient. In addition, it will greatly conflict with the long-term goal of monitoring outpatients at home. The sensor calibration for long-term outpatient monitoring requires some other practical and convenient alternative. 

As for the results on 18 TKA patients, the EPCA and BMI data analysis seemed to have no obvious correlation with the recovery progress. From the EPCA and BMI analysis results there are two potential approaches to obtain better results. The first one is to collect more data to perform statistical analysis on EPCA and BMI. The second approach is to conduct the clinical trial again and root out the inappropriate patient data before performing the analysis. For example, before analyzing the correlation with BMI, we should select the patients with comparable knee ROM before TKA. As shown in Figure 6, it is noteworthy that the pre-TKA knee ROM of the 18 patients are all above 115 degrees. According to the studies in [5,6], the 18 patients are still able to perform normal ADLs and walk, but with pain and loss of knee function. This also indicates that pre-filtering the patient’s pre-TKA knee ROM on a consistent basis is essential for the purpose of obtaining distinguishable analysis results. 

The results on analysis of the recovery of knee ROM by using different hemostatic agents showed that systemic transamin gave better results than intra-articular transamin. As we know the hemostatic agents are to inhibit the function of plasmin so as to reduce the blood loss from the surgery. The use of hemostatic agents might be indirectly related with the wound recovery and pain. Therefore, it further indirectly resulted in knee recovery progress and was reflected in the knee ROM results. 

As discussed in Section 3, there are still many challenges to overcome for achieving the goals of our research roadmap. Rehabilitation monitoring for TKA patients is a dynamic monitoring process that involves interactive feedback and adjustment of the rehabilitation force. Furthermore, there are many issues on long-term monitoring mechanism for discharged TKA patients in their home environment. In the home environment, without the assistance of medical professionals, how can patients do the sensor calibration? How do patients confirm the stabilization of the worn sensors? These are all critical issues for long-term TKA knee ROM monitoring. 

In conclusion, this work proposed a system architecture based on the five design principles addressed in the Introduction. We performed a small scale data collection during the pre-operative and post-operative TKA period. The obtained knee ROM data is analyzed with respect to the knee ROM recovery progress by the factors of EPCA, BMI, and hemostatic agents. The results showed that the three factors were truly related with the recovery progress of TKA knee ROM. In the future work, large scale clinical trials should be able to provide more solid results for statistical analysis. Other external calibration approaches instead of the robotic arms will be required when considering the monitoring in the home environment. The improved sensor-worn methods also require further experiments for verification. Furthermore, there are still many puzzles requiring novel and intelligent solutions to complete the whole research roadmap. 

## Figures and Tables

**Table 1:** Related works on skeleton-based monitoring infrastructure.

Year	Authors	Sensors	Wireless	Analysis	Reference
2008	Favre et al.	1 Gyro + 1 Acce.	N	Knee Joint	[11]
2009	Liu et al.	2 Gyros + 1 Gyro+ Acce.	N	Gait	[12]
2009	Takeda et al.	3 Gyros + 1 Acce.	N	Gait	[13]
2011	Jovicic et al.	3 Acce. Units + 2 Flexible Goinometers	Y	Gait	[14]
2013	Tadano et al.	7 Sensor units (1 Gyro+ 1 Acce.)	Y	Gait	[15]
2014	Calliess et al.	3 Sensor units (1 Gyro+ 1 Acce. + 1 magnetometer)	Y	Knee Joint	[16]
2015	Feldhege et al.	2 Sensor units (1 Gyro + 1 Acce.)	N	Knee Joint	[17]
2015	Chen et al.	2 Sensor units (1 Gyro + 1 Acce.)	Y	Knee Joint	[18]

* Acce. = accelerometer.
